# Acupuncture Alleviates CUMS-Induced Depression-Like Behaviors by Restoring Prefrontal Cortex Neuroplasticity

**DOI:** 10.1155/2023/1474841

**Published:** 2023-05-02

**Authors:** Peng Li, Wenya Huang, Yiping Chen, Muhammad Shahzad Aslam, Wenjing Cheng, Yang Huang, Wenjie Chen, Yanxun Huang, Xinnan Wu, Yining Yan, Junliang Shen, Tao Tong, Shuqiong Huang, Xianjun Meng

**Affiliations:** ^1^Department of Traditional Chinese Medicine, School of Medicine, Xiamen University, Xiamen, Fujian, China; ^2^Yueyang Hospital of Integrated Traditional Chinese and Western Medicine, Shanghai University of Traditional Chinese Medicine, Shanghai, China; ^3^Institute of TCM-Related Comorbid Depression, Nanjing University of Chinese Medicine, Nanjing, China; ^4^Second Clinical College, Shanxi University of Traditional Chinese Medicine, Taiyuan, Shanxi, China; ^5^School of Traditional Chinese Medicine, Xiamen University Malaysia, Sepang, Malaysia; ^6^Department of Traditional Chinese Medicine, School of Life Science, Xiamen University, Xiamen, Fujian, China; ^7^Shenzhen Research Institute of Xiamen University, Shenzhen, China

## Abstract

**Purpose:**

To explore the therapeutic efficiency of acupuncture and the related molecular mechanism of neural plasticity in depression.

**Methods:**

Chronic unpredictable mild stress- (CUMS-) induced rats were established for the depression animal model. There were a total of four rat groups, including the control group, the CUMS group, the CUMS+acupuncture group, and the CUMS+fluoxetine group. The acupuncture group and the fluoxetine group were given a 3-week treatment after the modeling intervention. The researcher performed the open-field, elevated plus maze, and sucrose preference tests to evaluate depressive behaviors. The number of nerve cells, dendrites' length, and the prefrontal cortex's spine density were detected using Golgi staining. The prefrontal cortex expression, such as BDNF, PSD95, SYN, and PKMZ protein, was detected using the western blot and RT-PCR.

**Results:**

Acupuncture could alleviate depressive-like behaviors and promote the recovery of the neural plasticity functions in the prefrontal cortex, showing the increasing cell numbers, prolonging the length of the dendrites, and enhancing the spine density. The neural plasticity-related proteins in the prefrontal cortex, including BDNF, PSD95, SYN, and PKMZ, were all downregulated in the CUMS-induced group; however, these effects could be partly reversed after being treated by acupuncture and fluoxetine (*P* < 0.05).

**Conclusion:**

Acupuncture can ameliorate depressive-like behaviors by promoting the recovery of neural plasticity functions and neural plasticity-related protein upregulation in the prefrontal cortex of CUMS-induced depressed rats. Our study provides new insights into the antidepressant approach, and further studies are warranted to elucidate the mechanisms of acupuncture involved in depression treatment.

## 1. Introduction

Depression is a common chronic neurological disease with typical manifestations in modern society, including low self-esteem, helplessness, and anhedonia [1, 2]. According to the latest analysis of the World Health Organization's survey, more than 300 million people have suffered from severe depression worldwide and the growth rate was about 18% in the last decade [3, 4]. Moreover, depression contributes to devastating mental trauma, brings pathophysiological disorders, and enhances susceptibility to some systematic diseases, such as cerebrovascular and cardiovascular diseases [5, 6]. Unfortunately, there are no completely effective therapeutic approaches for depression to date; the currently available antidepressant strategies such as medications, psychotherapies, or others have limited efficacy and significant side effects during the treatment period [7]. Therefore, it is profoundly significant to investigate the pathophysiology of depression, developing new approaches and insights for clinical practice.

Previous studies have demonstrated that depression can induce long-term neural plasticity changes in some specific brain regions, and the recovery of neurogenesis and synaptic plasticity are strongly related to the prognosis of depressive patients [8–10]. Several meta-analyses identified that acupuncture is more effective and safer than antidepressants. Moreover, researchers have scientifically proven acupuncture to be one of the effective therapies for depression, which was frequently applied in China, the US, the UK, Japan, and Korea [11]. Acupuncture is a vital component of Oriental medicine, and it has been proven to promote the impairment of neural plasticity and relieve the symptoms of depression patients [12–14]. Besides, acupuncture combined with antidepressant medication has an early onset of action and is safe and well tolerated, and the treatment combination appears to result in greater therapeutic efficacy than the medication therapy alone [15]. However, the specific underlying mechanism of acupuncture in depression treatment remains elusive.

The prefrontal cortex (PFC) is the part of the limbic cortex, involved in many stress-sensitive psychological disorders due to its rich neural interconnectivity [16, 17]. Brain-derived neurotrophic factor (BDNF), a plasticity-related protein, is widely distributed in the central nervous system [18, 19]. BDNF plays a critical role in the survival and differentiation of neurons during the development of the central nervous system, regulating neurogenesis and neuroplasticity [20, 21]. Postsynaptic density 95 (PSD-95) and synaptophysin (SYP) are two downstream synapse marker proteins of BDNF, relating to synaptic signal transmission [22]. It has proved that protein kinase M zeta (PKMZ), an atypical protein kinase C subtype, can mediate depressive-like behaviors in the prefrontal cortex of the depressed rat model, associating with the morphology of neural dendritic spines [23]. Acupuncture can interfere with the progression of depression by ameliorating neural plasticity; however, the relationship between acupuncture and neural plasticity-related proteins in the prefrontal cortex is still unknown.

In this study, we intend to explore the antidepressant mechanism underlying acupuncture based on the neuroplasticity and its neural plasticity-related proteins BDNF/PSD95/SYN/PKMZ in the prefrontal cortex of chronic unpredicted mild stress- (CUMS-) induced depressed rats, providing prospective insights for the treatment of depression in the clinic.

## 2. Materials and Methods

### 2.1. Experimental Design and CUMS Procedure

**Figure 1:**
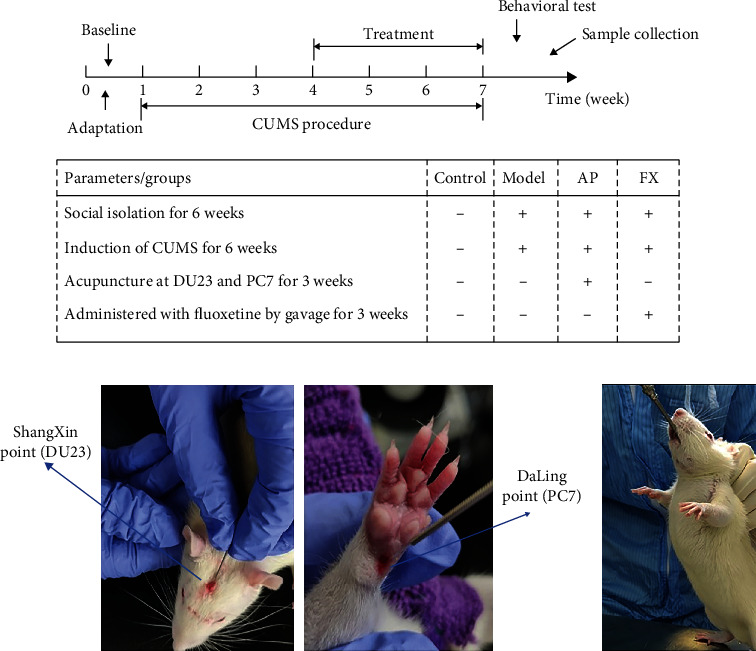
Experimental flowchart: (a) 1-week adaptation, 6-week CUMS procedure, 3-week treatment, behavioral test, and prefrontal cortex harvesting at the end of the experiment. Schematic diagram of acupuncture and fluoxetine operation in the rat. (b) Flat needling Shang xing (DU23) toward the direction of the nose; the needle was inserted as deep as 3–5 mm; obliquely stab Da ling (PC7) toward the direction of Nei guan (PC6); the needle was inserted as deep as 2–3 mm. (c) Fluoxetine solution (0.21 mg/ml) by gavage (1 ml/100 g).

Male Sprague-Dawley (SD) adults (grade SPF, weighing 120–150 g) were purchased from the Shanghai Slake Laboratory Animal Co., Ltd. with the license number SCXK (Shanghai) 2017-0005. Thirty-two (*n* = 32) rats were randomly divided into four groups with random number table: control group (Con), CUMS group (CUMS), acupuncture group (AP), and fluoxetine group (Fx). Rats in the control group were reared together, while rats in the other three groups were socially isolated by placing each rat in a single cage and undergoing CUMS procedure for six weeks. The acupuncture group and the fluoxetine group were then given 3 weeks of treatment during the latter 3 weeks of the modeling procedure (Figure 1). Fluoxetine is the first particular serotonin uptake inhibitor that helps alleviate the manifestations of depression in the clinic. According to previous literature, the depression model has been initially formed in 21 days (former 3 weeks). Based on the establishment of the initial model, we perform acupuncture and fluoxetine treatment while continuing the modeling procedure (latter 3 weeks). A similar protocol was present in our previous study [24]; according to the previous study [25], CUMS-induced depressed rats were induced by chronic unpredictable mild stress combined with single cage isolation for 42 days. We chose one stimulus every day, and the same stimulus could not occur continuously; the modeling procedure and schedule are shown in Table 1. Weight monitoring and behavioral test were conducted to estimate the state of the rats. The success criteria of the depressive model establishment showed a significant reduction in the behavioral score and body weight.

### 2.2. Acupuncture and Fluoxetine Intervention

**Figure 2:**
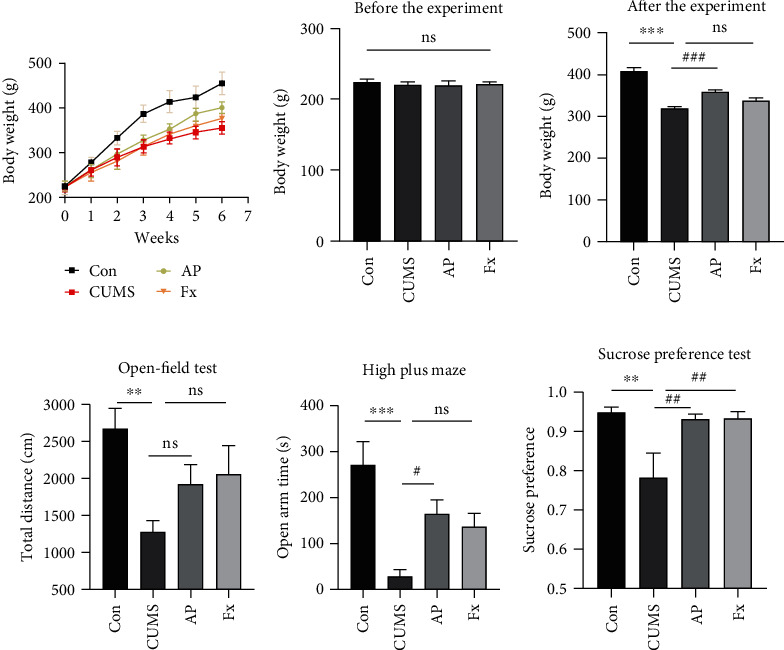
Body weight changes in all groups. (a) Comparison of rat's body weight growth trend among four groups during the whole experiment. (b) Body weight baseline before the experiment. (c) Body weight changes at the end of the experiment. Behavior changes in all groups. (d) Total distance in the open-field test among four groups. (e) Time in open arms of the elevated plus maze among four groups. (f) The percentage of sucrose consumed by the rat at sucrose concentrations of 1%. Compared with the control group, ^∗^*P* < 0.05, ^∗∗^*P* < 0.01, and ^∗∗∗^*P* < 0.001; compared with the CUMS group, ^#^*P* < 0.05, ^##^*P* < 0.01, and ^###^*P* < 0.001. Data represent means ± SEM (*n* = 8).

The procedure of acupuncture and fluoxetine operation in rats is shown in Figure 2(a). Briefly, rats in the acupuncture group were given acupuncture treatment at Shang xing (DU23) and Da ling (PC7) before daily CUMS stimulation. Acupuncture operation: flat needling Shang xing (DU23) toward the direction of the nose, the needle was inserted as deep as 3–5 mm; obliquely stab Da ling (PC7) toward the direction of Nei guan (PC6); the needle was inserted as deep as 2–3 mm. The needles used were sterilized disposable stainless-steel needles (0.18 mm diameter; Hanyi, Beijing, China) (Figure 2(a)). Then, the rats in the fluoxetine group were given fluoxetine (PHR1394-1G, Sigma-Aldrich) solution (0.21 mg/ml) by gavage (1 ml/100 g) before daily stimulation (Figure 2(b)).

### 2.3. Baseline and behavioral test

Rats' body weights were monitored once a week; the rat's mental state, eating status, activity level, and hair gloss were observed every day. Behavioral tests, including the open-field test (OFT), elevated plus maze test, and sucrose preference test, were executed at the end of the experiment.

OFT was executed to test the locomotor activity and depressive-like behaviors of rats; the operational steps were conducted as the previous study [26]. Each rat was singly placed into the center of an open box in which they could move freely, with walls and floor painted black (100 × 100 × 40 cm) and a fixed camera above the floor. The spontaneous exploration activity of rats during the 5-minute test was recorded by a webcam. The total distance and central residence time changes were assessed by the webcam as the criteria for depressive-like symptoms.

The elevated plus maze test was performed to estimate the anxiety-like behavior of depressive rats as reported in previous studies [27]. Rats were first placed in the center of the area and their heads facing the open arms. The EPM has two open arms (50 × 10 cm) and two closed arms (50 × 10 × 40 cm) connected with a central zone (10 × 10 cm). The autonomic activity of rats for crossing the open arms and the closed arms during the 5-minute test was recorded by a webcam fixed on top of the apparatus.

A sucrose preference test was executed to examine the anhedonia of rats; the operational steps were conducted as the previous study [28]. Water and food prohibition was performed in rats for 24 hours before the test. Each rat was given two bottles (one 1% sucrose and one pure water) which had been quantified beforehand; we removed bottles after 12 hours and calculated the liquid consumption. Sucrose preference (%) = [sucrose solution consumption/(sucrose solution consumption + pure water consumption)] × 100%.

The behavioral tests were conducted on days 0, 21, and 42 during the CUMS modeling period. Behavioral test on day 0 evaluated the baseline, and that on day 21 and day 42 monitored the behavioral status. The success criteria of the depressive model establishment are the significant reduction of behavioral scores, such as less total distance in the open-field test, less crossing the open arms in the elevated plus maze test, and less sucrose consumption in the sucrose preference test.

### 2.4. Enzyme-Linked Immunosorbent Assay

The prefrontal cortex and serum of rats was collected for ELISA. Prepare standard dilution solutions of corresponding concentrations in advance. The kits were operated following their manufacturer's instructions: TNF-*α* (JL17113, Jianglai Biology, Shanghai, China); CORT (H094, Jiancheng, Nanjing, China); and CRP (E-EL-R0506c), IL6 (E-EL-R0015c), CRH (E-EL-R0270c), and ACTH (E-EL-R0048c) (from Elabscience company in Wuhan, China). Each well's optical density value (OD value) was measured at a wavelength of 450 nm for 15 minutes, and the content was calculated according to the standard curve.

### 2.5. Golgi Staining

The prefrontal cortex of the rat was collected and fixed in 4% paraformaldehyde for more than 48 h. Cut the prefrontal cortex tissue into tissue blocks with a thickness of 2–3 mm according to the tissue site to be observed, gently rinse the brain tissue with normal saline several times, place in a 45 ml round bottom EP tube, add Golgi-Cox staining solution to submerge the brain tissue completely, and place in a cool and ventilated place and avoid light to treat for 14 days. A panoramic multilayer scanning conducted microscope inspection, image acquisition, and data analysis with a digital slice scanner. Analyses were performed as described previously [29]. The number of spinous cells was observed in the pyramidal neuron dendrites of the prefrontal cortex. Sholl analysis was used for dendritic tree examination [30]. The density of the spine was quantified, and the number of spines along the length was calculated [31].

### 2.6. Western Blot

The expressions of BDNF, PSD95, SYN, and PKMZ in the prefrontal cortex were detected by western blot. The methods were described previously [32]. Briefly, total protein was extracted from the brain tissue of rats with RIPA lysis buffer. The tissue lysates were centrifuged at 12,000 rpm for 10 min at 4°C, and the supernatants were separated for further analysis. SDS-PAGE gel with 10% or 12% according to protein molecular mass was prepared for the electrophoretic separation of protein samples and then transferred onto PVDF membranes. The membrane was blocked in Tris-buffered saline containing 5% nonfat milk and 0.1% Tween 20 for 2 h at room temperature and then incubated overnight with primary antibodies. After the membranes were washed with PBS, the secondary antibody was applied for 1 h at room temperature. After rinsing, the proteins were detected by enhanced chemiluminescence.

### 2.7. RT-PCR

The total RNA from rats' brain tissue (prefrontal cortex) was extracted using the TRIzol reagent. The detailed primer sequences for BDNF, PSD95, SYN, PKMZ, and the *β*-actin control are shown in Table 2. RNA then underwent reverse transcription followed by analysis using real-time PCR (RT-PCR). Finally, 2 *μ*l PCR products were detected by agarose gel. The electrophoresis conditions were 140 V, 15 min, gel imaging. We analyzed the gel using ImageJ software.

### 2.8. Statistical Analysis

All data were processed by GraphPad Prism 8.0 Windows software. The statistical data were expressed using mean ± standard deviation (*X* ± *s*). The variance was first tested for normality and homogeneity, and a one-way ANOVA was used for pairwise comparisons between groups that met the normal condition. If the homogeneity of variances is not met, then the Brown-Forsythe ANOVA test is selected. If the normality was not met, the rank-sum test was selected. Dunnett's T3 test was used for post hoc testing. The difference *P* < 0.05 was considered statistically significant.

## 3. Results

### 3.1. Acupuncture Alleviates the Manifestations of Depression in CUMS-Induced Rats

The body weight and behavior changes in different groups of CUMS-induced rats were analyzed. Our results showed that the bodyweight baseline of four groups including control, CUMS, acupuncture, and fluoxetine groups was similar before the specific treatment (*P* > 0.05) (Figure 2(b)), while with the progress of the experiment, the weight gain trend of the CUMS model rats all showed lower levels compared with the control rats (*P* < 0.001) (Figure 2(a)). As shown in Figure 2(c), CUMS-induced rats showed a significant reduction in body weight by comparing with the control group after the experiment (*P* < 0.001); the acupuncture group showed significant growth in body weight by comparing with the CUMS group (*P* < 0.001), while there are no significant differences in rats' body weight between the CUMS and fluoxetine groups (*P* > 0.05).

The results of the open-field test showed a significant reduction in the total distance of model rats compared to the control rats (*P* < 0.01); and the total distances showed an uptrend after being treated with acupuncture and fluoxetine without significant differences (*P* > 0.05) (Figure 2(d)). The results of the elevated plus maze test found that the open arm time was dramatically reduced in CUMS-induced rats (*P* > 0.001). The acupuncture treatment could improve the open arm time, and there was a significant elevation of open arm time in the acupuncture group, compared to the CUMS group (*P* < 0.05). The fluoxetine group showed an uptrend of the open arm time while without significant differences, comparing to the CUMS group (*P* > 0.05) (Figure 2(e)).

The sucrose preference test (SPT) examination showed a similar result. The 1% sucrose intake was significantly decreased in the CUMS group, whereas it was elevated in the acupuncture- and fluoxetine-treated groups (all *P* < 0.01) (Figure 2(f)).

### 3.2. Acupuncture Attenuates Abnormal Hyperactivity of the Prefrontal HPA Axis and Reduces Inflammation in CUMS Rats

**Figure 3:**
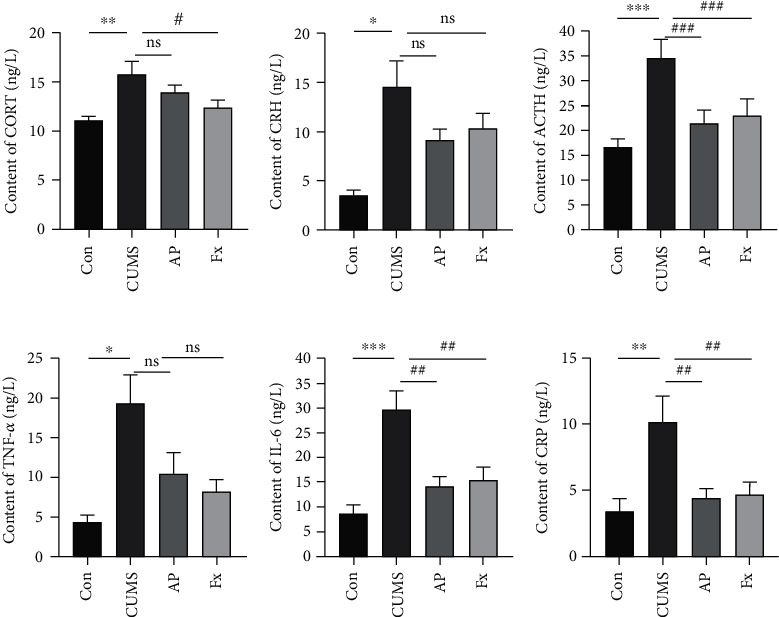
Expression of the Inflammatory factors. (a) CORT expression in serum. (b) CRH expression in serum. (c) ACTH expression in serum. (d) TNF-*α* expression in the prefrontal cortex. (e) IL-6 expression in the prefrontal cortex. (f) CRP expression in serum. Compared with the control group, ^∗^*P* < 0.05, ^∗∗^*P* < 0.01, and ^∗∗∗^*P* < 0.001; compared with the CUMS group, ^#^*P* < 0.05, ^##^*P* < 0.01, and ^###^*P* < 0.001. Data represent means ± SEM (*n* = 6).

Enzyme-linked immunosorbent assay was applied to check the expressions of the hypothalamic-pituitary-adrenal axis, including CORT, CRH, and ACTH in serum. As shown in Figures 3(a)–3(c), the expressions of CORT, CRH, and ACTH were all increased in the CUMS group (*P* < 0.01; *P* < 0.05; *P* < 0.001); however, these effects could be partly reversed after treatment by acupuncture (*P* > 0.05; *P* > 0.05; *P* < 0.001) and fluoxetine (*P* < 0.05; *P* > 0.05; *P* < 0.001) (Figures 3(a)–3(c)).

In addition, some inflammatory factor expressions of TNF-*α*, IL-6, and CRP were detected by ELISA as well. Similarly, the ELISA analysis represented that the inflammatory factor levels of TNF-*α* IL-6 in the prefrontal cortex and CRP in serum all increased in the CUMS group compared to the control group (*P* < 0.05; *P* < 0.001; *P* < 0.01), and after being treated with fluoxetine and acupuncture, the inflammatory factors of them were all downregulated (*P* > 0.05; *P* < 0.01; *P* < 0.01) (Figures 3(d)–3(f)).

### 3.3. Effects of Acupuncture on Neural Features in CUMS-Induced Rats

**Figure 4:**
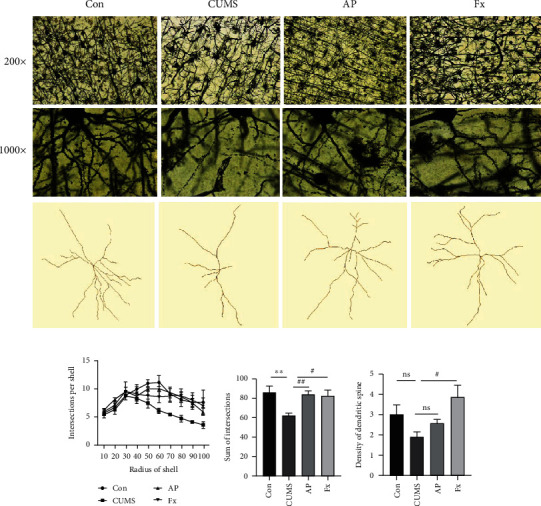
(a) Golgi staining of rats' prefrontal cortex (200x (up column) and 1000x (down column)). Morphological analyses of dendritic arborization and spine density of prefrontal cortex. (b) Intersection per shell of pyramidal neuron arborization. (c) Sum of intersections of dendrites. (d) Density of dendritic spines. Compared with the control group, ^∗^*P* < 0.05, ^∗∗^*P* < 0.01, and ^∗∗∗^*P* < 0.001; compared with the CUMS group, ^#^*P* < 0.05, ^##^*P* < 0.01, and ^###^*P* < 0.001. Data represent means ± SEM (*n* = 3).

Neuronal cells in the rat prefrontal cortex were stained using the Golgi method, and the staining images showed that the neuronal cells in the CUMS group were disorderly arranged with an unclear outline, decreased dendrite number, and shorter dendrite length, compared to its control group. After treatment by acupuncture, all the characteristics of neurons were relatively recovered, including increased cell numbers, prolonged length of the dendrites, and enhanced spine density; and the fluoxetine intervention also showed similar results (Figure 4(a)).

Further analyses on neuron morphological character and spine density in the prefrontal cortex were performed. The neuron images were processed by the Sholl function of ImageJ, observing the branches and length of dendrites, and then, the spine density of the prefrontal cortex was calculated. Our results showed that the intersection number per shell and the development of the dendritic spine were significantly impaired in the CUMS group, which could be reversed by acupuncture treatment (*P* < 0.01), and the fluoxetine intervention also showed similar results (*P* < 0.01) (Figures 4(b)–4(d)). As shown in Figure 4(d), the density of dendritic spines in the CUMS group was at a low level (*P* < 0.05); after the intervention treatments, dendritic spine density was at a higher level in the acupuncture group compared to the CUMS group, although there was no significant difference (*P* > 0.05); the fluoxetine group was at a higher level in dendritic spine density compared with the CUMS group (*P* < 0.05).

### 3.4. Acupuncture Promotes the Expressions of Neural Plasticity-Related Proteins in the Prefrontal Cortex of CUMS-Induced Rats

**Figure 5:**
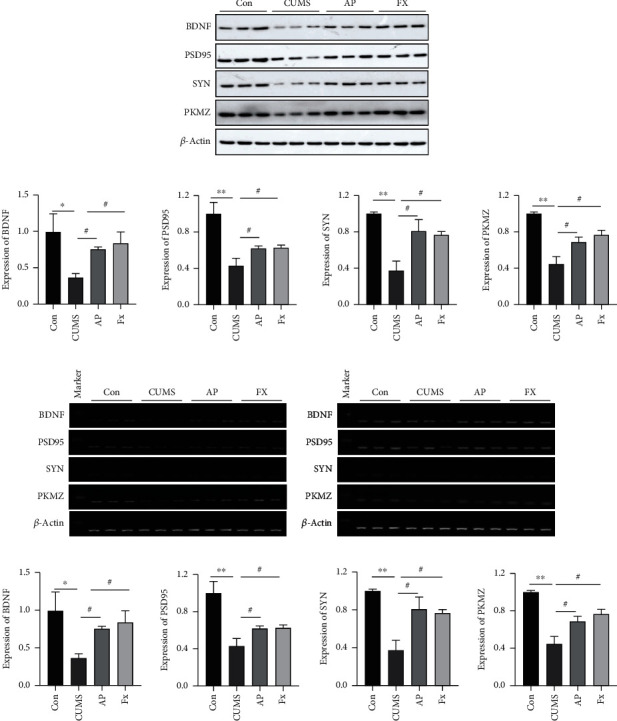
(a) The expression of neural plasticity-related proteins in the prefrontal cortex measured by western blot. (b) The mRNA level of neural plasticity-related proteins in the prefrontal cortex measured by RT-PCR. Compared with the control group, ^∗^*P* < 0.05, ^∗∗^*P* < 0.01, and ^∗∗∗^*P* < 0.001; compared with the CUMS group, ^#^*P* < 0.05, ^##^*P* < 0.01, and ^###^*P* < 0.001. Data represent means ± SEM (*n* = 3).

Western blot was applied to check the expressions of neural plasticity-related proteins, including BDNF, PSD95, SYN, and PKMZ, in the prefrontal cortex. As shown in Figure 5(a), the expressions of BDNF, PSD95, SYN, and PKMZ were all downregulated in the CUMS group; however, these effects could be partly reversed after treatment by acupuncture (*P* < 0.05) and the fluoxetine intervention also showed similar results (*P* < 0.05).

In addition, total mRNA expressions of BDNF, PSD95, SYN, and PKMZ were detected by RT-PCR as well. Similarly, the RT-PCR analysis represented that the mRNA levels of BDNF, PSD95, SYN, and PKMZ in the prefrontal cortex all decreased in the CUMS group compared to the control group. After being treated with acupuncture, their mRNA level was elevated (*P* < 0.05), and the fluoxetine intervention also showed a similar result (*P* < 0.05) (Figure 5(b)).

## 4. Discussion

Depression is a chronic and recurrent disease, with a high prevalence rate worldwide. Current available therapeutic approaches such as medications and psychotherapies have limited efficacy and obvious side effects [33, 34]. Recently, acupuncture, a traditional Chinese medicine therapy, has been proven effective and safe in depression treatment by several clinical meta-analyses [35]. Nevertheless, the underlying mechanism of acupuncture in depression treatment is still unclear. In the present study, we established the CUMS-induced depressed rats, and then, the animal model was identified based on the body weight and behavior changes. Our results demonstrated the depression-like behavior induced by CUMS procedure, while this behavior could be partly reversed by acupuncture treatment. Besides, acupuncture had a similar and safer antidepression therapeutic effect as fluoxetine intervention.

Acupuncture has been identified as a safe and effective alternative therapy for depression with fewer side effects. The choice of acupoints is crucial to ensure acupuncture's curative effects. Daling (PC7) is considered the original acupoint of the pericardium meridian. According to the meridian-collateral theory in Traditional Chinese Medicine, Daling (PC7) can not only relieve the fire evil enveloped by the heart but also has great efficacy in treating some mental diseases by calming the mind and benefiting the nerves. Shangxing (DU23) is located in the governor meridian, which has a role in supervising and awakening the mind. These two acupoints are essential for the therapeutic effectiveness of mental illness. Interestingly, we explored the repair effect of these two acupoints on depressive-like behavior in CUMS rats and found that the effect of PC7 is similar to that of DU23 [36]. Not only that, we also found that acupuncture at DU23 could adjust the IL-6 expression in CUMS rats [37]. Therefore, the acupoints of PC7 and DU23 were elected in our study to treat the depression model rats. The classic antidepressant medicine, fluoxetine, was used as a positive control group. There have been many studies about acupuncture's effect on depression. However, few studies clarified the possible molecular mechanisms of the hypothalamic-pituitary-adrenal axis based on acupuncture involved in depression treatment. In molecular biology, our study found that CUMS caused abnormal hyperactivity of the hypothalamic-pituitary-adrenal axis, whereas acupuncture and fluoxetine reversed this phenomenon and downregulated the expression of CORT, CRH, and ACTH. In addition, acupuncture also alleviated the level of inflammation in the prefrontal cortex of the CUMS group and decreased the expression of inflammatory factors such as TNF-*α*, IL-6. Interestingly, neuroinflammation and neuroplasticity were interrelated, with inflammation being a key node in the pathological changes of neuroplasticity.

In a neural network, neuronal cells are the fundamental functional units, helping integrate and transmit bioelectric signals through dendrites. The information processing way of neuronal cells is the dynamic process in response to emotional changes, injury, external stimulators, and so forth [38]. The structural and functional changes of neural cells in response to various stimuli are known as neural plasticity, including depression. Previous studies have demonstrated that the biological mechanism of depression is associated with neural plasticity in specific brain areas, especially the prefrontal cortex [39]. The functional synapses in the central nervous system were disarranged in depression patients [40] by emotional disorder and stress. It has been observed that chronic and repetitive stress can aggravate the manifestations of depression through the impairment of neural plasticity. Antidepressant treatment could help release the manifestations by enhancing neural plasticity [41]. Similarly, in this study, we found that the arrangement of neural cells was disordered in the prefrontal cortex of CUMS-induced depressed rats, with specific features of unclear boundary, decreased number of dendrites, and shorter length of dendrites by Golgi staining. Moreover, the morphological results of dendritic arborization and spine density were impaired in the prefrontal cortex of CUMS rats as well. Intriguingly, acupuncture or fluoxetine treatment could significantly reverse these detrimental effects, which provide a new insight into potential acupuncture applications in depression treatment.

Complex molecular protein interactions regulate the synaptic formation and neural plasticity [42]. The abnormal expression levels of functional proteins are closely related to depression susceptibility, including brain-derived neurotrophic factor (BDNF), postsynaptic density 95 (PSD-95), synaptophysin (SYN), and protein kinase M zeta (PKMZ). BDNF can regulate neural plasticity and synaptic transmission by increasing Ca2+ levels and mitochondrial movement in neurons. BDNF enhances the release of neurotransmitters to protect neuroplasticity and promotes cannabinoid-mediated neurogenesis [43]. As a scaffold of synaptic components required for synapse development, PSD-95 is the main organizer of signal complexes on the postsynaptic membrane. The inhibition of PSD-95 can severely affect postsynaptic function and plasticity [44]. SYN is a weak donor at the presynaptic terminal, regulating the activity-dependent synapses [45]. Moreover, PKMZ has been confirmed to play roles in learning and long-term potentiation, which could also promote neurite shaft protrusion [46]. In the present study, the total protein and mRNA levels of BDNF, PSD95, SYN, and PKMZ were detected in the prefrontal cortex of the CUMS-induced rat model. It is indicated that both the protein and mRNA levels of BDNF, PSD95, SYN, and PKMZ were decreased in the CUMS group, while these effects can be partly eliminated by acupuncture or fluoxetine treatment. These molecular results were consistent with prior morphological results stained by Golgi.

Altogether, CUMS-induced rats can be served as a reliable depression animal model, and acupuncture is effective and safe for ameliorating depression-like behavior, increasing cells number, prolonging the length of the dendrites, and enhancing the spine density in CUMS-induced rats. Besides, acupuncture treatment can upregulate the expressions of neural plasticity-related proteins in the prefrontal cortex including BDNF, PSD95, SYN, and PKMZ. Our study provides new perspectives on the antidepressant approach and clarifies its related molecular mechanisms. Further studies are warranted to elucidate the mechanisms of acupuncture involved in depression treatment.

## 5. Conclusion

**Figure 6:**
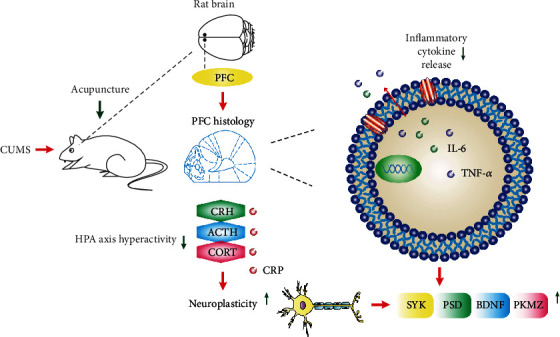
Schematic diagram of the mechanism by which acupuncture alleviates depression-like behavior in CUMS rats. Acupuncture at Shang xing (DU23) and Feng fu (DU16) points can inhibit the hyperactivity of the HPA axis and improve the level of inflammation and restore neuroplasticity in depressed rats.

In this study, we revealed that acupuncture can play an antidepressant effect in CUMS-induced depression model rats. Acupuncture at Shang xing (DU23) and Feng fu (DU16) points can inhibit the hyperactivity of the HPA axis, improve the level of inflammation, and restore the neuroplasticity of the prefrontal cortex of depressed rats, which may be achieved by increasing the abundance of neuronal dendritic spines and related protein expression (Figure 6). In conclusion, based on our current study, it can be concluded that acupuncture, one of the most widely used complementary and alternative therapies, is closely related to the modulation of neuroplasticity in the field of exerting antidepressants.

## Figures and Tables

**Table 1:** CUMS modeling procedure.

Stress	Day
Fasting (24 h)	1, 13, 19, 32, 42
45°C hot water stimulation (5 min)	2, 10, 24, 34, 41
Restraint (3 h)	3, 17, 20, 30, 40
Water deprivation (24 h)	4, 14, 25, 28, 39
Day and night inversion (24 h)	5, 11, 21, 36, 38
4°C cold water stimulation (5 min)	6, 18, 26, 35, 37
Squirrel cage tilt (45°, 24 h)	7, 15, 22, 33, 36
Noise stimulation (3 h, 80 dB)	8, 12, 27, 31
Tail clamping (3 min)	9, 16, 23, 29

**Table 2:** PCR primer information sheet.

mRNA primers	Sequence (5′-3′)	Product length (bp)
Rat-*β*-actin-F	CTGGCTCCTAGCACCATGAA	180
Rat-*β*-actin-R	AAAACGCAGCTCAGTAACAGTC	
Rat-*β*-BDNF-F	GCCTCCTCTGCTCTTTCT	141
Rat-*β*-BDNF-R	GCCGTTACCCACTCACTA	
Rat-*β*-PSD95-F	ACACCCATTGCCCAGAAC	142
Rat-*β*-PSD95-R	TCTCCACGCAGTCATAAAG	
Rat-*β*-SYP-F	ACAGCAGTGTTCGCTTTCA	209
Rat-*β*-SYP-R	CAGAGCACCAGGTTCAGG	
Rat-*β*-PKMZ-F	CACATTAAGCTGACGGACTA	
Rat-*β*-PKMZ-R	TCTCAAACATAAGGACACCC	166

## Data Availability

The RNA sequences can be freely obtained in this article. The data that support the results of this study are available on request from the corresponding author, Xianjun Meng.

## References

[1] Hobzova M., Prasko J., Vanek J. (2017). Depression and obstructive sleep apnea. *Neuro Endocrinology Letters*.

[2] Incze M. A. (2019). I’m worried about depression-what should I know?. *JAMA Internal Medicine*.

[3] James S. L., Abate D., Abate K. H. (2018). Global, regional, and national incidence, prevalence, and years lived with disability for 354 diseases and injuries for 195 countries and territories, 1990-2017: a systematic analysis for the Global Burden of Disease Study 2017. *The Lancet*.

[4] Barnett R. (2019). Depression. *The Lancet*.

[5] Zhang Y., Chen Y., Ma L. (2018). Depression and cardiovascular disease in elderly: current understanding. *Journal of Clinical Neuroscience*.

[6] Duman R. S., Aghajanian G. K., Sanacora G., Krystal J. H. (2016). Synaptic plasticity and depression: new insights from stress and rapid- acting antidepressants. *Nature Medicine*.

[7] Liu W., Ge T., Leng Y. (2017). The role of neural plasticity in depression: from hippocampus to prefrontal cortex. *Neural Plasticity*.

[8] Singhal G., Baune B. T. (2017). Microglia: an interface between the loss of neuroplasticity and depression. *Frontiers in Cellular Neuroscience*.

[9] Doan L., Manders T., Wang J. (2015). Neuroplasticity underlying the comorbidity of pain and depression. *Neural Plasticity*.

[10] Calabrò M., Mandelli L., Crisafulli C. (2018). Neuroplasticity, neurotransmission and brain-related genes in major depression and bipolar disorder: focus on treatment outcomes in an Asiatic sample. *Advances in Therapy*.

[11] Li M., Niu J., Yan P. (2020). The effectiveness and safety of acupuncture for depression: an overview of meta-analyses. *Complementary Therapies in Medicine*.

[12] Liu C. Z., Kong J., Wang K. (2017). Acupuncture therapies and neuroplasticity. *Neural Plasticity*.

[13] Liu C. Z., Chen J. D., Zhang M. (2018). Advances on the acupuncture therapies and neuroplasticity. *Evidence-based Complementary and Alternative Medicine*.

[14] Tong P., Dong L. P., Yang Y., Shi Y. H., Sun T., Bo P. (2019). Traditional Chinese acupuncture and postpartum depression: a systematic review and meta-analysis. *Journal of the Chinese Medical Association*.

[15] Chan Y. Y., Lo W. Y., Yang S. N., Chen Y. H., Lin J. G. (2015). The benefit of combined acupuncture and antidepressant medication for depression: a systematic review and meta-analysis. *Journal of Affective Disorders*.

[16] Hultman R., Mague S. D., Li Q. (2016). Dysregulation of prefrontal cortex-mediated slow-evolving limbic dynamics drives stress-induced emotional pathology. *Neuron*.

[17] Wood M., Adil O., Wallace T. (2019). Infralimbic prefrontal cortex structural and functional connectivity with the limbic forebrain: a combined viral genetic and optogenetic analysis. *Brain Structure & Function*.

[18] Kowiański P., Lietzau G., Czuba E., Waśkow M., Steliga A., Moryś J. (2018). BDNF: a key factor with multipotent impact on brain signaling and synaptic plasticity. *Cellular and Molecular Neurobiology*.

[19] Leal G., Comprido D., Duarte C. B. (2014). BDNF-induced local protein synthesis and synaptic plasticity. *Neuropharmacology*.

[20] Wang B., Ning H., Reed-Maldonado A. B. (2017). Low-intensity extracorporeal shock wave therapy enhances brain-derived neurotrophic factor expression through PERK/ATF4 signaling pathway. *International Journal of Molecular Sciences*.

[21] Lu B., Nagappan G., Lu Y. (2014). BDNF and synaptic plasticity, cognitive function, and dysfunction. *Handbook of Experimental Pharmacology*.

[22] Wang T., Weng H., Zhou H. (2022). Esketamine alleviates postoperative depression-like behavior through anti-inflammatory actions in mouse prefrontal cortex. *Journal of Affective Disorders*.

[23] Yan W., Liu J. F., Han Y. (2020). Correction: protein kinase M*ζ* in medial prefrontal cortex mediates depressive-like behavior and antidepressant response. *Molecular Psychiatry*.

[24] Li P., Huang W., Yan Y. N. (2021). Acupuncture can play an antidepressant role by regulating the intestinal microbes and neurotransmitters in a rat model of depression. *Medical Science Monitor*.

[25] Wang Q., Timberlake M. A., Prall K., Dwivedi Y. (2017). The recent progress in animal models of depression. *Progress in Neuro-Psychopharmacology & Biological Psychiatry*.

[26] Hu C., Luo Y., Wang H. (2017). Re-evaluation of the interrelationships among the behavioral tests in rats exposed to chronic unpredictable mild stress. *PLoS One*.

[27] Walia V., Garg C., Garg M. (2019). NO-sGC-cGMP signaling influence the anxiolytic like effect of lithium in mice in light and dark box and elevated plus maze. *Brain Research*.

[28] Liu M. Y., Yin C. Y., Zhu L. J. (2018). Sucrose preference test for measurement of stress-induced anhedonia in mice. *Nature Protocols*.

[29] García-Rojo G., Fresno C., Vilches N. (2017). The ROCK inhibitor fasudil prevents chronic restraint stress-induced depressive-like behaviors and dendritic spine loss in rat hippocampus. *The International Journal of Neuropsychopharmacology*.

[30] Gibb R., Kolb B. (1998). A method for vibratome sectioning of Golgi-Cox stained whole rat brain. *Journal of Neuroscience Methods*.

[31] Ferreira T. A., Blackman A. V., Oyrer J. (2014). Neuronal morphometry directly from bitmap images. *Nature Methods*.

[32] Wang Y., Jiang H., Meng H. (2017). Antidepressant mechanism research of acupuncture: insights from a genome-wide transcriptome analysis of frontal cortex in rats with chronic restraint stress. *Evidence-based Complementary and Alternative Medicine*.

[33] Berton O., Nestler E. J. (2006). New approaches to antidepressant drug discovery: beyond monoamines. *Nature Reviews. Neuroscience*.

[34] Khin N. A., Chen Y. F., Yang Y., Yang P., Laughren T. P. (2011). Exploratory analyses of efficacy data from major depressive disorder trials submitted to the US Food and Drug Administration in support of new drug applications. *The Journal of Clinical Psychiatry*.

[35] Armour M., Smith C. A., Wang L. Q. (2019). Acupuncture for depression: a systematic review and meta-analysis. *Journal of Clinical Medicine*.

[36] Cheng W. J., Li P., Huang W. Y. (2021). Acupuncture relieves stress-induced depressive behavior by reducing oxidative stress and neuroapoptosis in rats. *Frontiers in Behavioral Neuroscience*.

[37] Chen Y., Hao C., Chen W. (2022). Anti-depressant effects of acupuncture: the insights from NLRP3 mediated pyroptosis and inflammation. *Neuroscience Letters*.

[38] Howard D. M., Adams M. J., Shirali M. (2018). Genome-wide association study of depression phenotypes in UK Biobank identifies variants in excitatory synaptic pathways. *Nature Communications*.

[39] Jensen M., Overgaard M. (2011). Neural plasticity and consciousness. *Frontiers in Psychology*.

[40] Hall F. S., Li A., Li B. (2018). Neural plasticity in mood disorders. *Neural Plasticity*.

[41] Yu H., Zou Z., Zhang X. (2018). Inhibition of phosphodiesterase 4 by FCPR03 alleviates lipopolysaccharide-induced depressive-like behaviors in mice: involvement of p38 and JNK signaling pathways. *International Journal of Molecular Sciences*.

[42] Amtul Z., Ur R. A. (2015). Neural plasticity and memory: molecular mechanism. *Reviews in the Neurosciences*.

[43] Ferreira F. F., Ribeiro F. F., Rodrigues R. S., Sebastião A. M., Xapelli S. (2018). Brain-derived neurotrophic factor (BDNF) role in cannabinoid-mediated neurogenesis. *Frontiers in Cellular Neuroscience*.

[44] Fossati G., Morini R., Corradini I. (2015). Reduced SNAP-25 increases PSD-95 mobility and impairs spine morphogenesis. *Cell Death and Differentiation*.

[45] Kwon S. E., Chapman E. R. (2011). Synaptophysin regulates the kinetics of synaptic vesicle endocytosis in central neurons. *Neuron*.

[46] Chihabi K., Morielli A. D., Green J. T. (2016). Intracerebellar infusion of the protein kinase M zeta (PKM*ζ*) inhibitor zeta-inhibitory peptide (ZIP) disrupts eyeblink classical conditioning. *Behavioral Neuroscience*.

[47] Huang W., Li P., Cheng W. J. (2021). Acupuncture promoted the recovery of the neural plasticity functions and the repair of neural plasticity-related proteins in the prefrontal cortex of CUMS-induced depressed rats. *Research Square*.

